# Epidermal Growth Factor Receptor Mutation and Anaplastic Lymphoma Kinase Gene Fusion: Detection in Malignant Pleural Effusion by RNA or PNA Analysis

**DOI:** 10.1371/journal.pone.0158125

**Published:** 2016-06-28

**Authors:** Yi-Lin Chen, Chung-Ta Lee, Cheng-Chan Lu, Shu-Ching Yang, Wan-Li Chen, Yang-Cheng Lee, Chung-Hsien Yang, Shu-Ling Peng, Wu-Chou Su, Nan-Haw Chow, Chung-Liang Ho

**Affiliations:** 1 Molecular Diagnostics Laboratory, Department of Pathology, National Cheng Kung University Hospital, Tainan, Taiwan; 2 Department of Medical Laboratory Science and Biotechnology, National Cheng Kung University Hospital, Tainan, Taiwan; 3 College of Medicine, Molecular Medicine Core Laboratory, Research Center of Clinical Medicine, National Cheng Kung University Hospital, Tainan, Taiwan; 4 The Institute of Basic Medical Sciences, College of Medicine, National Cheng Kung University Hospital, Tainan, Taiwan; 5 The Institute of Molecular Medical, College of Medicine, National Cheng Kung University Hospital, Tainan, Taiwan; 6 Division of Hematology/Oncology, Department of Internal Medicine, Tainan Municipal Hospital, Tainan, Taiwan; 7 Yuan’s General Hospital, Kaohsiung, Taiwan; 8 The Association of Medical Technologists, Tainan, Taiwan; University of Navarra, SPAIN

## Abstract

Analyzing *EGFR* mutations and detecting *ALK* gene fusion are indispensable when planning to treat pulmonary adenocarcinoma. Malignant pleural effusion (MPE) is a devastating complication of lung cancer and sometimes the only source for mutation analysis. The percentage of tumor cells in the pleural effusion may be low; therefore, mutant enrichment is required for a successful analysis. The *EGFR* mutation status in MPE was determined using three methods: (1) PCR sequencing of genomic DNA (direct sequencing), (2) mutant-enriched PCR sequencing of genomic DNA using peptide nucleic acid (PNA-sequencing), and (3) PCR sequencing of cDNA after reverse transcription for cellular RNA (RNA-sequencing). RT-PCR was also used to test cases for *ALK* gene fusion.

PNA-sequencing and RNA-sequencing had similar analytical sensitivities (< 1%), which indicates similar enrichment capabilities. The clinical sensitivity in 133 cases when detecting the common *EGFR* exon 19 and exon 21 mutations was 56.4% (75/133) for direct sequencing, 63.2% (84/133) for PNA-sequencing, and 65.4% (87/133) for RNA-sequencing. RT-PCR and sequencing showed 5 cases (3.8%) with *ALK* gene fusion. All had wild-type *EGFR*. For *EGFR* analysis of MPE, RNA-sequencing is at least as sensitive as PNA-sequencing but not limited to specific mutations. Detecting *ALK* fusion can be incorporated in the same RNA workflow. Therefore, RNA is a better source for comprehensive molecular diagnoses in MPE.

## Introduction

Of the 1.5 million new cases of lung cancer every year, about 85% are non-small-cell lung cancer (NSCLC), the leading cause of cancer death worldwide.[1] Approximately 70–80% of patients with NSCLC present with advanced disease and a poor prognosis.[1] There are many driver mutations in NSCLC, among which the *epidermal growth factor receptor* (*EGFR*) mutation and *ALK* fusion are the 2 most common therapeutic targets.[2]

The *EGFR* gene is mutated in a considerable proportion of primary lung adenocarcinomas, especially in patients who respond to gefitinib and erlotinib, which are small-molecule *EGFR* tyrosine kinase inhibitors (TKIs).[3] Patients with sensitive *EGFR* mutations were recommended for first-line TKI treatment. Both the response rate (RR) and progression-free survival (PFS) of patients with *EGFR* mutations who were treated with TKIs were better than those of patients treated with chemotherapy.[4,5] In contrast, patients with wild-type *EGFR* did not benefit from *EGFR* TKI treatment. Therefore, determining the *EGFR* status is paramount for *EGFR* TKI treatment. The *EGFR* mutation frequency is about 22–64% in Asia[6] and 9–21% in western countries.[2,7] It is more frequent in non-smokers and East Asians.[6] However, smokers can have a positive rate as high as 30%. Therefore, no clinical parameter can be used to pre-select patients for *EGFR* TKI treatment.[6] It is recommended that all patients with NSCLC have their *EGFR* gene status determined before TKI therapy. In addition, detecting somatic mutations of exons 19 and 21 in *EGFR* may provide prognostic information for patients with advanced NSCLC.[8,9] Therefore, the *EGFR* mutation is both a prognostic factor and a predictive factor for the response of TKI targeting therapy.

A fusion protein between the N-terminal portion of the echinoderm microtubule-associated protein-like 4 (*EML4*) protein and the intracellular signaling portion of *ALK*[10] tyrosine kinase receptor, which has recently[11] been identified in a small subset of patients with NSCLC, is now recognized as an important oncogenic driver in NSCLC. Multiple *EML4-ALK* variants in NSCLC have been identified, all containing the same C-terminal kinase domain with a gain of function.[12] The incidence of the *EML4-ALK* fusion gene in NSCLC is around 3–5% with no significant differences between Asian and western countries.[13,14] Currently, three different techniques are available for detecting *ALK* rearrangement: fluorescence in situ hybridization (FISH), reverse transcription polymerase chain reaction (RT-PCR), and immunohistochemistry (IHC), each with advantages and limitations.[15] RT-PCR is a highly sensitive technique that can define both the fusion partner and the variant of *ALK* gene fusion.

Malignant pleural effusion (MPE) is a common and devastating complication of NSCLC, especially adenocarcinoma. In some patients, MPE is an initial manifestation and thus an easy path for collecting malignant cells for molecular investigation.[16–19] Sampling MPE is generally repeatable and safe. Therefore, sampling the MPE of NSCLC is a practical approach for investigating *EGFR* mutations, especially when patients present with advanced and unresectable NSCLC.[20–23] There are several testing methods available for mutational analysis in pleural fluid, including PCR direct sequencing, mutant-enriched PCR, pyrosequencing, and real-time PCR. The sensitivity of PCR direct sequencing is about 10–20%.

Enrichment methods are required because the tumor cells in the effusion may coexist with copious background inflammatory cells and mesothelial cells. To ensure sufficient cancer cells for analysis, the enrichment is usually done using macro-dissection, manual micro-dissection, or laser-capture micro-dissection of the smear or cell blocks.[24]

Supplementary techniques that target specific mutations have been developed, however, such as the peptide nucleic acid (PNA) approach to improve mutation detection (PNA-sequencing).[25–29] The rationale for using these technologies is that approximately 90% of *EGFR* mutations are either exon 19 deletion (exon 19 del) or L858R in exon 21.[9] On this base, PNA-sequencing on common mutations was developed to meet clinical needs.[9]

A recent cohort study [16] reported using RNA as a favorable source for analyzing *EGFR* mutations in MPE. It was believed that mutant *EGFR* mRNA from tumor cells was enriched in the RNA samples from pleural effusions because the non-tumor cells produced little wild-type *EGFR* mRNA.[16]

The aim of this study was to assess the sensitivity of *EGFR* mutation analysis using direct sequencing, PNA-sequencing, and RNA-sequencing of MPE in lung adenocarcinoma.

## Materials and Methods

### Patients

Effusion with abnormal cytology (malignancy, suspicious, atypical) was centrifuged and the cell pellet was stored for molecular testing. From January 2011 to June 2015, there were 160 molecular tests for pleural effusion ordered at our hospital. Their cytology diagnoses included: 157 “malignancy”, 2 “suspicious for malignancy”, and 1 “atypical cells”. Eighty-five of the 157 malignant cases had subtyping information in the diagnoses: 71 adenocarcinoma and 14 NSCLC not otherwise specified (NSCLC-NOS). Forty-one cases had biopsy tissue proof, all of which were adenocarcinoma. One hundred forty-two cases had enough cells for both DNA and RNA tests, 16 for RNA tests only, and 2 for DNA tests only. The 142 cases were used for methodological comparison. Seventy-three of the 142 malignant cases had subtyping information in the diagnoses, including 62 adenocarcinoma and 11 NSCLC-NOS. The RNA test covers exons 18–21 of *EGFR*; the PNA tests for exon 19 del and L858R in exon 21 have good performance and cover approximately 90% of all *EGFR* mutations. Other mutations are much less cost-effective if PNA were used. Because our PNA test was not designed to detect mutations in exons 18 and 20, we excluded 9 such cases. All 9 patients were diagnosed by RNA-sequencing, including five exon 18 mutations (1 G709A, 3 G719A, and 1 G719S) and 4 exon 20 mutations (2 p.Ala767_Val769 dup, 1 p.Ser768_Asp770 dup, and 1 p.Val769_Asp770 ins CG). The remaining 133 cases contained either wild-type *EGFR* or the common mutations (exon 19 del or L858R).

This study was approved by National Cheng Kung University Hospital’s Institutional Review Board (IRB #: B-BR-101-137). All patients signed a written informed consent form for molecular analysis.

Demographic and clinical information of these patients was recorded: age, sex, smoking history, tumor staging, performance status (Eastern Cooperative Oncology Group performance status (ECOG PS),[30] treatment regimens, and maximal response. Patients who had smoked fewer than 100 cigarettes in their lifetime were categorized as *never smokers*, and those who had smoked more than 100 cigarettes were categorized as *former smokers*. The response to gefitinib was evaluated in accordance with Response Evaluation Criteria in Solid Tumors (RECIST) guidelines.[31–33]

### Specimen collection and preparation

Pleural effusion was collected in sterile tubes. Samples (15 mL) were centrifuged at 250 × *g* for 10 min, and the cell pellet was stored in a reagent (RNAlater; Qiagen, Hilden, Germany) at −80°C until use. If the cellularity was high enough, samples were also submitted for DNA extraction. Both RNA and DNA for molecular analysis were extracted in approximately 89% (142/160) of the MPEs.

### Extracting genomic DNA and RNA from cell pellets

One kit (QIAmp DNA Mini Kit; Qiagen) was used to extract genomic DNA from cell pellets, and another (RNeasy; Qiagen) to extract RNA from stored cell pellets.

### PCR sequencing of cDNA from cell-derived RNA (RNA-sequencing)

The reverse transcriptase (RT)-PCR was done using reverse transcriptase (Superscript II; Invitrogen, Carlsbad, CA) as previously described.[34] Exons 18–21 of the *EGFR* were amplified using specific primer sets (Table 1). The amplicons (557 bp) were purified and sequenced using a kit (BigDye Terminator Sequencing Kit; Applied Biosystems, Foster City, CA). The sequencing products underwent electrophoresis using ABI 3500 genetic analyzer (ABI Prism 3500; Applied Biosystems). The sequences were compared with the *EGFR* sequences in the GeneBank from the National Center for Biotechnology Information (NCBI) (accession number NM_005228.3). Both forward and reverse sequences were analyzed, and the original chromatograms were manually examined by two medical technologists.

**Table 1:** PCR primers and the PNA sequences.

**PCR primer (DNA)**	
Exon 19 forward	5′-AAC GTC TTC CTT CTC TCT CTG TCA T-3′
Exon 19 reverse	5′-AGC AGG GTC TAG AGC AGA GCA GCT GCC-3′
Exon 21 forward	5′-ATC TGT CCC TCA CAG CAG GGT C-3′
Exon 21 reverse	5′-GGC TGA CCT AAA GCC ACC T-3′
**Sequencing primer (DNA)**	
Exon 19 forward	
Exon 19 reverse	5′-AGC AGG GTC TAG AGC AGA GCA GCT GCC-3′
Exon 21 forward	5′-CCC TCA CAG CAG GGT C-3′
Exon 21 reverse	5′-GAC CTA AAG CCA CCT C-3′
**PCR primer (RNA)**	
Exons 18–21 forward	5′-GGA GCC TCT TAC ACC CAG TG-3′
Exons 18–21 reverse	5′-TGC CTC CTT CTG CAT GGT AT-3′
**Sequencing primer (RNA)**	
Exons 18–21 forward	5′-GCC TCT TAC ACC CAG TGG AG-3′
Exons 18–21 reverse	5′-TGC CTC CTT CTG CAT GGT AT-3′
**PNA (Peptide Nucleic Acid)**	
Exon 19	5′-AGA TGT TGC TTC TCT TAA-3′
Exon 21 (L858R)	5′-CAG TTT GGC CA^a^G CCC A-3′

^a^Subsequent nucleotide is a locked nucleic acid.

### Direct sequencing and PNA-sequencing using cell-derived genomic DNA

Exons 18, 19, 20, and 21 of the *EGFR* gene were amplified using culture-independent nested PCR (Table 1) as previously described.[17,21,35] For PNA-sequencing, PNA is used to construct the PCR clamp reactions; the PNA clamp suppresses the amplification of wild-type DNA, thereby increasing the preferential amplification of the mutant sequences. PNA oligos were synthesized by Bio-Synthesis, Inc. (Lewisville, TX). The mutant-enriched PCR was done in a mixture of PCR primers (10 μM each), PNA oligo (100 μM each), genomic DNA (25 ng), and a DNA polymerase (Super Therm Gold Master Mix; Bionovas Biotechnology, Toronto, Ontario, Canada). The PCR amplicons were purified and then subjected to bidirectional sequencing.

### PCR amplification

The PCR was done using a thermal cycler (G-Storm; GMI, Inc., Ramsey, MN). The cycling was as follows: 95°C for 6 min, 35 cycles of 95°C for 30 s, 60°C for 1 min, and 72°C for 1 min. Nested PCR for *EGFR* mutation analysis was done using the same reaction conditions.

### Constructing plasmid clones of *EGFR* exons 19 and 21 sequences and of mixed cell lines

The pGEMT easy vector plasmids, which contain *EGFR* exon 19 del (E746_A750 del) or L858R mutations, were derived from human primary NSCLC tissue using PCR cloning. The sequence of each plasmid was confirmed using Sanger sequencing. To evaluate the sensitivity of PNA-sequencing, plasmid DNAs containing *EGFR* mutants were serially diluted with genomic DNA from A549 cells with wild-type *EGFR*. To determine the detection limit of *EGFR* mutations at the cellular level, genomic DNA with E746_A750 del was extracted from H1650 cells and mixed with genomic DNA from A549 cells (wild-type *EGFR*) in ratios ranging from 1:1 to 1:100. The A549 and H1650 cell lines were obtained from the American Type Culture Collection (Manassas, VA, USA). Similarly, to determine the detection limit of *EGFR* mutations at the RNA level, total RNA from H1650 and A549 cells was mixed in ratios ranging from 1:1 to 1:100.

### RT-PCR quality control

For quality control, two primer pairs were used to amplify the *GAPDH* (165 bp) and *β2-microglobulin* (256 bp) genes from 25 ng of each sample.[36] Primers for *GAPDH* were: GAA GGT GAA GGT CGG AGT C and TGG AAT TTG CCA TGG GTG GA; primers for *β2-microglobulin* were: TGG AGG CTA TCC AGC GTA CT and CGG CAG GCA TAC TCA TCT TT. The final concentrations of the primers were 0.8 μM in a 25-μL reaction. PCR conditions were as follows: 95°C for 30 s, followed by 35 cycles each of 95°C for 30 s, 60°C for 30 s, and 72°C for 30 s with a final 10-min extension at 72°C. Gel electrophoresis was done using a 2% agarose gel.

### Detecting the *EML4*-*ALK* fusion gene using multiplex PCR

The procedure for *EML4*-*ALK* multiplex PCR was adapted from Sanders et al.[14] and Soda et al.,[11] using Super Therm Gold Master Mix (Bionovas) in one tube. The primers used are shown in Table 2. The most common fusion types of *KIF5B-ALK* were detected using the primers in Table 2. Reaction conditions were: one cycle of 95°C for 5 min, followed by 35 cycles of 95°C for 30 s, 60°C for 30 s, 72°C for 30 s, and then one cycle of 72°C for 10 min. RT-PCR amplicons were purified and sequenced using a kit (Big Dye Terminator Sequencing Kit; Applied Biosystems). The different variants were determined and then compared with previously published reports.

**Table 2:** Primers used for multiplex PCR gene fusion detection.

Gene and primers	Location
***EML4*-*ALK* detection**	
5′-AAG ATC ATG TGG CCT CAG TG-3′	Forward primer: *EML4* exon 2
5′-CTG CAG ACA AGC ATA AAG ATG-3′	Forward primer: *EML4* exon 6
5′-GAC TCA GGT GGA GTC ATG C-3′	Forward primer: *EML4* exon 13
5′-CCA GGA CAC TGT GCA GAT TT-3′	Forward primer: *EML4* exon 17
5′-CAG ATA TGG AAG GTG CAC TG-3′	Forward primer: *EML4* exon 20
5′-TCT TGC CAG CAA AGC AGT AGT TGG-3′	Reverse primer: *ALK* exon 20
***KIF5B-ALK* detection**	
5′-TCA AGC ACA TCT CAA GAG CAA GTG-3′	Forward primer: *KIF5B* exon 2
5′-GAC AGT TGG AGG AAT CTG TCG ATG-3′	Forward primer: *KIF5B* exon 17
5′-CAG CTG AGA GAG TGA AAG CTT TGG-3′	Forward primer: *KIF5B* exon 24

### Statistical analysis

Patient characteristics (sex, tumor histology, and smoking habit) were tabulated in relation to the mutation status. Fisher’s exact test was used to analyze associations between patient characteristics and the presence of *EGFR* mutations. Significance was set at *p* < 0.05 (two-sided). SPSS 17.0 for Windows (SPSS Inc., Chicago, IL) was used for all analyses.

### Evaluating the response to *EGFR*-TKI

The tumor response to *EGFR* TKI was evaluated using chest X-rays of the disease sites every 2–4 weeks, and computed tomography[30] every 8–12 weeks after treatment. Treatment responses were assessed according to the Response Evaluation Criteria in Solid Tumors (RECIST) using the unidimensional method.[37,38]

## Results

### Sensitivity assays using cloned *EGFR* DNA fragments

**Fig 1:**
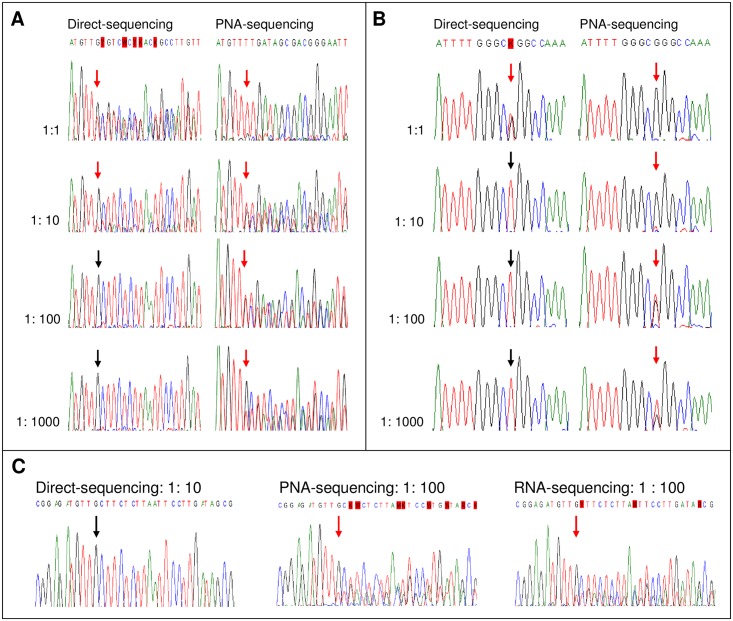
Sensitivity assays using cloned DNA fragments or cell lines. Cloned DNA fragments containing the (A) *EGFR* E746_A750 del or (B) *EGFR* L858R mutation were serially diluted in genomic DNA from A549 cells with wild-type *EGFR*. Direct sequencing of PCR products (direct sequencing; left column) and mutant-enriched PCR sequencing using peptide nucleic acid (PNA-sequencing; right column) were done to determine their analytical sensitivities. The arrows indicate the detected mutation sites. (C) Genomic DNA or total RNA from H1650 cells (E746_A750 del) and A549 cells (wild-type *EGFR*) were mixed in ratios ranging from 1:1 to 1:100. The DNA underwent direct sequencing and PNA-sequencing. The RNA was underwent PCR sequencing of cDNA after reverse transcription (RNA-sequencing). The arrows indicate the detected mutation sites. The sequencing chromatograms from the lowest detectable ratios are shown in the panel.

To determine the sensitivity of PNA-sequencing, cloned DNA fragments (mutants) were serially diluted in genomic DNA from A549 cells (wild-type *EGFR*) with various calculated gene copy numbers. The analytical sensitivity of PNA-sequencing was estimated to be less than (better than) 1% for both E746_A750 del (Fig 1A) and L858R mutations (Fig 1B). Consistent with general experiences with frame-shift mutations, PCR direct sequencing showed a sensitivity of 10% for E746_A750 del (Fig 1A).

### Sensitivity assays using cell lines with *EGFR* mutations

We used mixed cell lines to estimate the sensitivities at the cellular level. Genomic DNA from H1650 cells (E746_A750 del) and A549 cells (wild-type *EGFR*) was mixed in ratios ranging from 1:1 to 1:100. Consistent with the above assay using cloned DNA fragments, the analytical sensitivity of PNA-sequencing for E746_A750 del was estimated to be < 1% at the cellular level (Fig 1C). For RNA-sequencing, complementary DNA was obtained using RT-PCR sequencing. The sensitivity of RNA-sequencing was similar to that of the PNA-sequencing at the cellular level (< 1%) (Fig 1C).

### Detecting *EGFR* mutations using direct-, PNA- and RNA-sequencing

The analytical sensitivities of PNA-sequencing and RNA-sequencing met our criterion for *EGFR* mutation analysis (< 1%). They were subsequently used for routine clinical testing. PCR direct sequencing was also used as a companion test to PNA-sequencing. If a mutation was detected using any one of the 3 methods, a positive report was issued. From January 2011 to June 2015, there were 160 molecular tests done for pleural effusion; 101 were positive for *EGFR* mutations (63%).

One hundred forty-two of the 160 cases had enough cells for both DNA and RNA tests. The mean age of the patients was 69.0 years (range: 31–100 years). *EGFR* mutation was detected in 96 cases (67.6%). *EGFR* mutations were not associated with sex (men: 30.9% versus women: 36.6%; *p* = 0.275) and smoking history (37.3% of never smokers versus 7.0% of former or current smokers; *p* = 0.321), but associated with age≥ 65 (*p* = 0.008). (S1 Table).

### Comparing the three detection methods

Because the PNA-sequencing method used in this study detects only exon 19 del and L858R mutations, we excluded 9 cases with other mutations for the comparison. In the remaining 133 cases, direct sequencing detected *EGFR* mutations in 75 cases (56.4%), PNA-sequencing detected *EGFR* mutations in 84 cases (63.2%), and RNA-sequencing detected *EGFR* mutations in 87 cases (65.4%) of MPE. DNA direct sequencing detected 32 cases with exon 19 deletions and 43 with exon 21 L858R mutations. PNA-sequencing detected 36 cases with exon 19 deletions, 47 cases with L858R mutations, and one case with double mutations (E745_A750 del and L858R). RNA-sequencing detected 39 cases with exon 19 deletions and 48 cases with L858R mutations (Table 3).

**Table 3:** Distribution of *EGFR* Exon 19 and 21 mutations detected using the three methods.

	Direct sequencing	PNA-sequencing	RNA-sequencing
**Total cases (n = 133)**			
Wild-type (%)	58 (43.6)	49 (36.8)	46 (34.6)
Mutation type (%)	75 (56.4)	84 (63.2)	87 (65.4)
**Detected as a single mutation**			
Exon 19	32	36	39
Exon 21	43	47	48
**Detected as double mutations**			
Del+L858R		1^a^	

^a^DNA: WT, PNA: Del+L858R, RNA: L858R

### Discrepancy using different detection methods

Parallel analysis showed that RNA-sequencing and PNA-sequencing were significantly (*p* < 0.0001) correlated (Table 4).

**Table 4:** Comparison of PNA-sequencing and RNA-sequencing in 133 MPE samples.

**Method**		**RNA-sequencing**	
		WT	MT
**PNA-sequencing**	WT	46	3^a^
	MT	0	84

WT: wild-type; MT: mutant type;

^a^Del 19, L858R; Significance set at *p* < 0.0001.

### Clinical response to *EGFR*-TKIs

**Fig 2:**
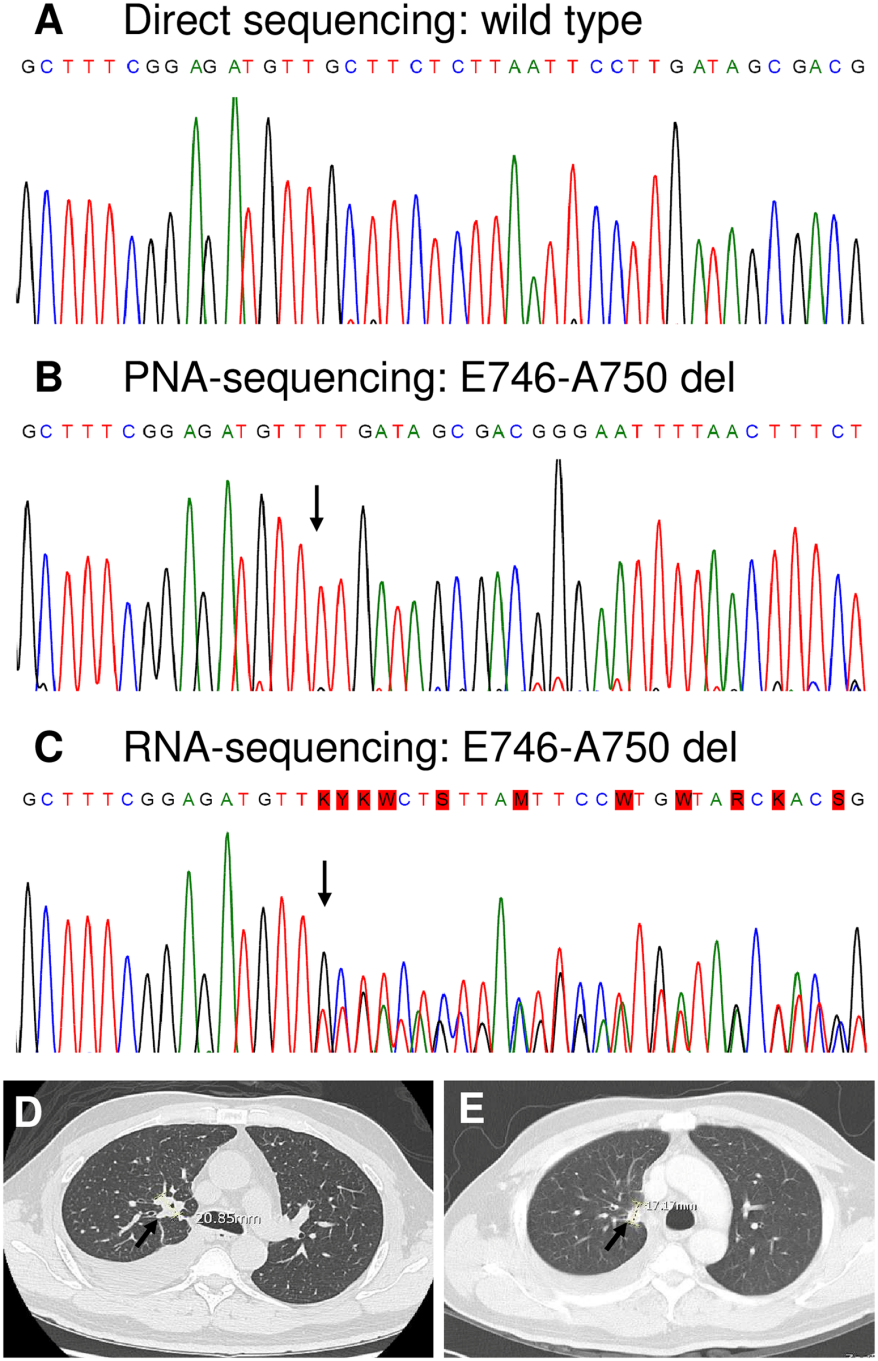
Example of a case of detected *EGFR* exon 19 mutation using PNA- and RNA-sequencing but not direct sequencing. (A-C) The mutation (*EGFR* exon 19 E746_A750 del) in the cellular component of MPE was detected using PNA- and RNA-sequencing but not direct sequencing. The arrow indicates a mutation. Wild-type sequence: GCTTCTCTTAATTCCTT; mutant sequence: TTGATAGCGACGGGAAT. Pretreatment (D) and post-treatment (E) CT images. The tumor size decreased from 2.1 cm to 1.7 cm (19%) after 18 months of TKI treatment.

Forty-seven of the 87 patients with *EGFR* mutations (exon 19 deletions or L858R) received TKIs treatment in our hospital. The clinical decisions were all based on *EGFR* mutations detected in MPE. The best clinical responses according to RECIST after TKIs treatment were: 15 partial response (32%), 22 stable disease (47%), and 10 progressive disease (21%). Fig 2 shows one patient as an example. The MPE was tested negative by DNA-sequencing (Fig 2A), but RNA and PNA-sequencing showed exon 19 E746_A750 del (Fig 2B and 2C). The tumor shrank from 2.1 to 1.7 cm (19%) after 18 months of TKI treatment (Fig 2D and 2E).

### Multiplex RT-PCR for detecting *ALK* gene fusion

**Fig 3:**
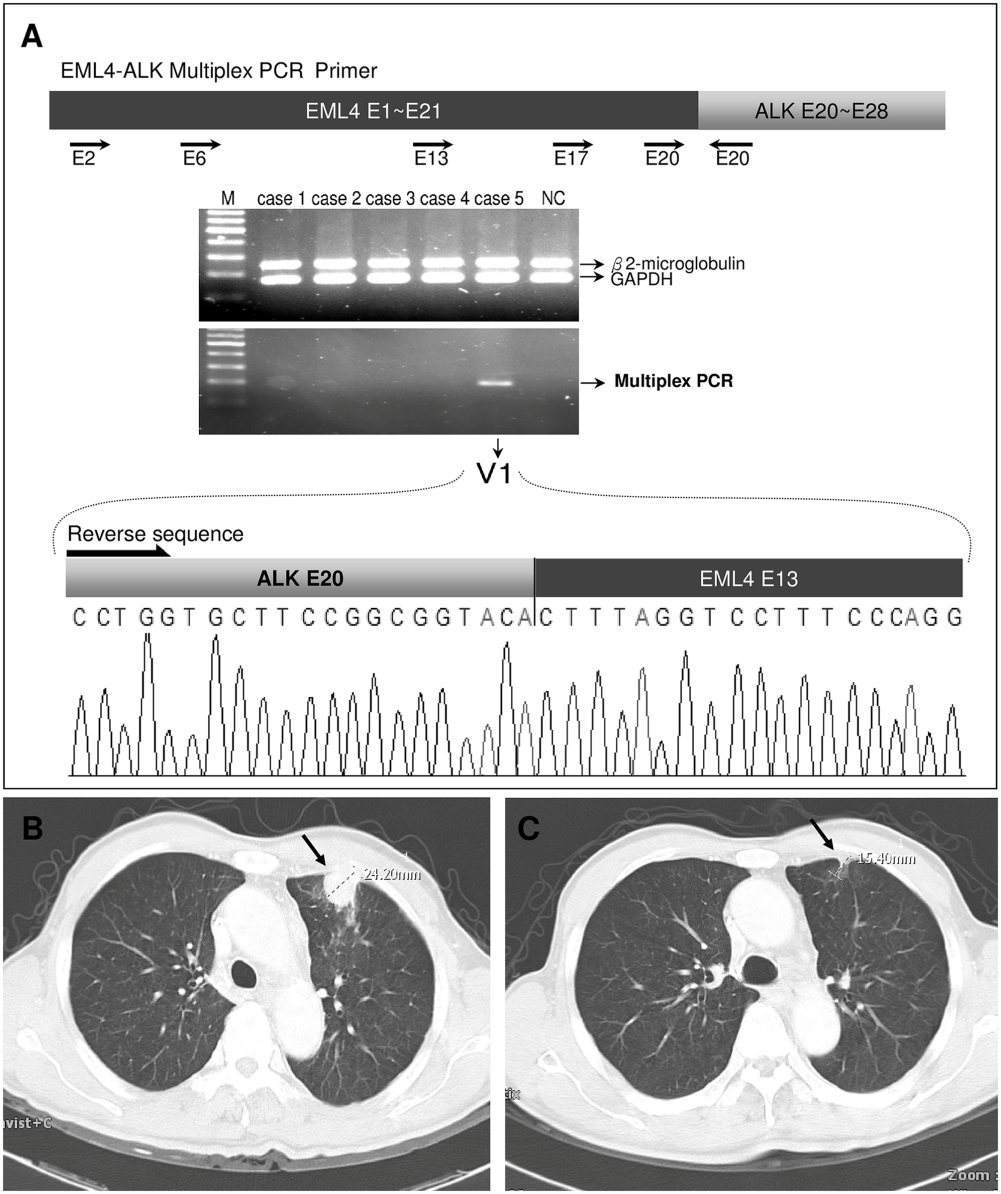
Example of a case of detected *EML4*-*ALK* fusion using multiplex PCR. The RT-PCR product was sequenced and confirmed to be variant 1 (E13; A20) of *EML4*-*ALK* fusion. Pretreatment (B) and post-treatment (C) CT images. The tumor size decreased from 2.4 cm to 1.5 cm (36%) after 6 months of TKI treatment.

Multiplex RT-PCR, which was then used to test all 113 cases for *EML4-ALK* and *KIF5B-ALK* gene fusion, detected that five of 46 (10.8%) wild-type *EGFR* cases had *EML4-ALK* fusion. All of them were variant 1 (E13; A20), which was confirmed using Sanger sequencing (Fig 3). The 67 cases with exon 19 del or L858R mutations showed negative results.

### Clinical response to *ALK*-TKIs

Two of the 5 patients with *ALK* fusions received Ceritinib treatment in our hospital. The clinical decisions were based on *ALK* Fluorescent in Situ Hybridization performed on tissue biopsy following the detection of *ALK* fusion in MPE. The best clinical responses according to RECIST after TKIs treatment were: 1 partial response and 1 progressive disease. Fig 3 shows the *EML4-ALK* fusion gene sequencing and the CT images of the patient with partial response. The tumor shrank from 2.4 to 1.5 cm (36%) after 6 months of TKI treatment (Fig 3B and 3C).

## Discussion

The traditional method for *EGFR* mutation analysis is direct-sequencing, a technique in which non-malignant cells are likely to interfere with in a heterogeneous tissue sample.[24,39] Tsai et al.[16] reported that RNA-sequencing was significantly more sensitive for detecting *EGFR* mutations (67.3% vs. 44.7%). Consistent with other studies,[16] in 133 cases, we detected only 75 (56.4%) exon 19 del and L858R mutations using direct-sequencing compared with 87 (65.4%) using RNA-sequencing. Such a result is compatible with the notion that RNA-sequencing is more sensitive than direct-sequencing (using DNA) because of the natural enrichment of the mutant *EGFR* mRNA in the cells of pleural effusion.

We found that RNA-sequencing was slightly more sensitive than PNA-sequencing in clinical samples (65.4% vs. 63.2%, respectively). This is because the sensitivity of RNA-sequencing is determined by the total *EGFR* expression level, not by the number of background cells (mesothelial and inflammatory cells). In contrast, the sensitivity of DNA-based PNA-sequencing depends solely upon the number of background cells. The tumor cells have the mutant genome and they express the mutant *EGFR* mRNA. The background cells within MPEs have considerably lower *EGFR* expression in comparison with the overexpression of *EGFR* in NSCLC cells. Because PNA does not completely (100%) block the wild-type background signal, all of the genomic DNA of the background cells will contribute to wild-type signals in PNA-sequencing. However, if the background cells express a very low level of *EGFR*, the mutant *EGFR* expressed by the tumor cells will be highly enriched in RNA-sequencing, no matter how many background cells there are. Using H1650 as a background, PNA-sequencing and RNA-sequencing had similar sensitivities (Fig 1). It is possible that the background cells in MPE have even lower *EGFR* expression than do H1650 cells, which makes the RNA-sequencing more sensitive than PNA-sequencing in clinical samples.

We found that RNA-sequencing might reveal discrepancies between gene mutations in genomic DNA and in mRNA. PNA-sequencing found double mutations (E745_A750 del and L858R) in one case in this study, which RNA-sequencing showed as only singly mutated at L858R. Whether two common sensitive mutations (E745_A750 del and L858R) can reside in the same *EGFR* molecule is an interesting question.

The sensitivities of *EGFR* mutation detection were 56.4% for direct-sequencing, 63.2% for PNA-sequencing, and 65.4% for RNA-sequencing. The detection rates in our study were higher than those in Hung et al.,[20] who reported a 41% (12/29) detection rate using direct-sequencing, but comparable to those in Tsai et al.,[16] who reported a 67.3% detection rate using RNA-sequencing. Altogether, *EGFR* mutation in the MPE of lung adenocarcinoma is generally between 60% and 70% in Taiwan, regardless of the patient’s sex, smoking status, and age. Additional prospective studies are needed to confirm our observation.

Among the 133 patients, 22 had available formalin fixed paraffin embedded (FFPE) tissue with amplifiable DNA by Qiagen Therascreen^®^ EGFR RGQ PCR Kit. Nineteen of the 22 results were consistent with those of MPE (7 wild-type and 12 mutants). The remaining 3 (2 wild-type and 1 exon 19 del single mutation) were inconsistent. The corresponding results from MPE were: p. Glu746_Pro753 del Val, Ser (c.2237_2257>TCT), p. L747_T751 del ins Asn (c.2239_2253>AAT), and exon 19 del + T790M double mutations. The first 2 were less common exon 19 deletions which were not included in the 29 mutations to be detected by the Therascreen^®^ kit. This demonstrated the ability of RNA-sequencing and/or PNA-sequencing for the detection of uncommon mutations.

Among the 46 EGFR wild-type cases, 9 had available FFPE tissue for immunohistochemistry. All results were consistent between MPE and FFPE tests (3 positive, 6 negative).

When clinically detecting *EGFR* mutations, several things need to be considered. For PNA-sequencing, the only modification is to add PNA to the PCR mixture. No additional cost for instruments is required. The technology is simple, rapid, and cost-effective for detecting specific mutant DNA sequences. For RNA-sequencing, careful handling to avoid ribonuclease (RNase) activity is critical for successful testing. Commercially available RNA stabilizing reagents together with refrigeration can minimize RNA degradation. Actually, fusion gene testing based on RT-PCR is a routine procedure for leukemia in many molecular laboratories and should not prohibit using RNA to test the cellular component of pleural effusions. However, the workflow of sample collection and transport should be streamlined to avoid RNA degradation. RNA is an ideal material for detecting fusion genes, not only for *ALK*, but also for *ROS1* and *RET* found in lung adenocarcinoma.[40] The subtypes of gene fusions can be identified using RT-PCR and Sanger sequencing. It is conceivable that different subtypes respond differently to different TKIs. Such additional benefits may justify the workflow changes for RNA testing. The most sensitive method for *EGFR* and *ALK* testing might be true only when the testing specimen is pleural effusion. Generally, RNA-based techniques are not practical for general diagnosis, especially in reference laboratories that receive samples from many centers.

*EGFR* mutations and *ALK* gene fusion were thought to be mutually exclusive events in lung adenocarcinoma. Our finding that *ALK* gene-fusion-positive cases are all negative for *EGFR* mutations might support this notion. However, evidence that the co-occurrence of the two driver mutations is not uncommon, and that such cases might benefit from dual treatments with both *EGFR* and *ALK* TKIs, has recently been reported.[41] When treated with a single TKI, *ALK* TKI might be a better choice than *EGFR* TKI.[41] It is highly likely that in the near future, comprehensive molecular diagnoses for lung adenocarcinoma will include both *EGFR* and *ALK* testing. *EGFR* mutation analysis alone may not be adequate, because a patient with a sensitive *EGFR* mutation might have a concomitant *ALK* fusion, which might require *ALK* TKI for optimal treatment.

In conclusion, we have provided evidence that supports MPE as a practical specimen for molecular analysis in patients with NSCLC. RNA-sequencing is more sensitive than PNA-sequencing for detecting *EGFR* mutations for targeted therapy. PNA-sequencing can be used as an alternative when RNA quality is not optimal. We suggest RNA-sequencing for *EGFR* mutations and RT-PCR for *ALK* gene fusion for comprehensive molecular testing of NSCLC MPE.

## Supporting Information

S1 Table Patient characteristics and frequency of *EGFR* mutations. (PDF)
