# Supplementary material for: Epidermal Growth Factor Receptor Mutation and Anaplastic Lymphoma Kinase Gene Fusion: Detection in Malignant Pleural Effusion by RNA or PNA Analysis
Source: PLoS One. 2016 Jun 28;11(6):e0158125. doi: 10.1371/journal.pone.0158125 (PMC4924845; doi:10.1371/journal.pone.0158125)
Supplement: S1 Table — (PDF) [file pone.0158125.s001.pdf]

S1 Table. Patient characteristics and frequency of *EGFR* mutations

Characteristic	No. of patients	Mutation Type	Wild Type	Mutation Rate (%)	*p*
**Sex**					
Male	73	44	29	30.9	
Female	69	52	17	36.6	NS
**Age (years)**					
< 65	55	36	19	25.3	
≥ 65	87	60	27	42.2	0.008
**Smoking status**					
Never-smoker	71	53	18	37.3	
Former or current smoker	12	10	2	7.0	
Not available	59	33	26	23.2	NS

NS: not significant (i.e., $p > 0.05$ [two-sided]).
